# Another Piece of the Ionic Liquid’s Puzzle: Adsorption of Cl^–^ Ions

**DOI:** 10.1021/acs.jpcc.3c07991

**Published:** 2024-01-31

**Authors:** Liis Siinor, Heigo Ers, Piret Pikma

**Affiliations:** Institute of Chemistry, University of Tartu, Ravila 14A, 50411 Tartu, Estonia

## Abstract

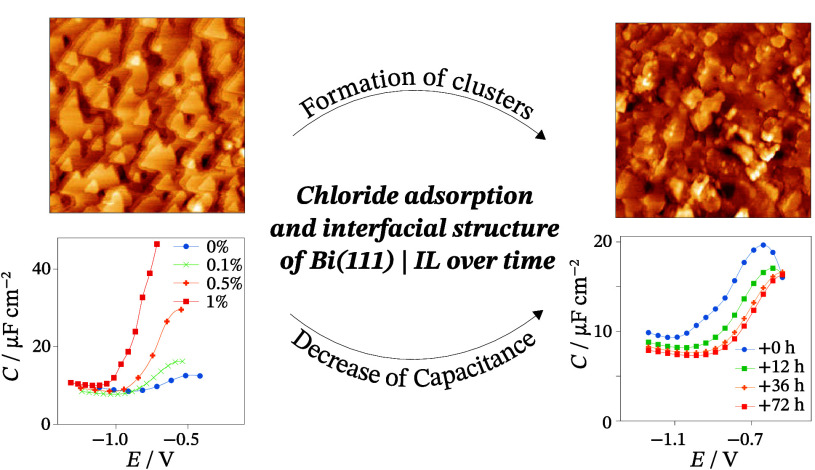

Classical electrochemical and microscopy methods were used to characterize the interfacial processes of the adsorption of chloride ions from ionic liquids at the Bi(111) single crystal electrode. The mixture of 1-ethyl-3-methylimidazolium tetrafluoroborate and 1-ethyl-3-methylimidazolium chloride was electrochemically characterized by using cyclic voltammetry and electrochemical impedance spectroscopy. *In situ* scanning tunneling microscopy images showed the formation of superstructures at the electrode’s surface over an extended period of time. The specific adsorption of chloride ions reaches an equilibrium state in a more viscous ionic liquid medium slower than in aqueous and organic solvents. Capacitance values increase considerably (also depending on alternative current frequency) at the potential region, where the specific adsorption of chloride ions with partial charge transfer occurs.

## Introduction

Interactions and processes that determine the interfacial structure and properties of the electrode|ionic liquid (IL) interface are crucial for developing fundamental understanding and various applications.^1−6^ This implies that the expected progress of electrochemical energy storage devices and other appliances relies on the success of describing these interfacial systems and processes in detail. Various factors define the interfacial properties of any electrode|electrolyte system.^7−9^ These factors can be roughly divided into 3 groups: electrode-related (the effect of surface structural and electronic properties), electrolyte-related (the chemical composition and/or presence of additives), and external factors (applied potential, temperature, etc.). Numerous well-recognized research groups have described the properties of the electrode|ionic liquid interface. The consensus is that ILs form ordered interfacial layers at electrodes, while the interfacial processes are much slower than in other electrolytes.^10−12^ Despite the consensus, numerous fundamental questions have yet to be answered.

Ionic liquids have been a subject of interest for the wider scientific community for a few decades. ILs have modifiable physical and chemical properties due to their chemical composition and structural variability. This allows the derivation of suitable cation and anion combinations for the specific research question or possible application.^13,14^ Among other excellent properties, ILs are salts with considerable liquid ranges, remarkable electrochemical stability, and low vapor pressure.^15^ At the same time, the ILs electrolyte conductivities are lower than expected due to the strong correlations between the ions, evident from the high viscosities and low diffusion coefficients.^16−18^ ILs have been studied as promising electrolytes for energy conversion devices and in industrial applications as reaction media, solvents, lubricants, and performance additives.^19−25^ Along with common ILs, polymerized ILs have attracted notable interest.^26^ In these ILs, the properties of IL monomers (e.g., high ionic conductivity and thermal stability) are integrated into the polymers. Breakthroughs by applying polymerized ILs can be expected in biochemical and medical applications, CO_2_ capture, sorption, and gas separation. Furthermore, ionic liquid crystals are advanced electrolytes with remarkable safety and electrochemical performance that enable the feasibility of design and the manipulation of defined ion transport channels through modulated nanosegregated structures.^27^ Therefore, ILs have a significant role in developing modern sustainable energy storage devices and transitioning to a sustainable chemistry-based society.

Typically, ILs are prepared from dialkylimidazolium or alkylpyridinium halide salts (mostly chloride).^28^ Thus, trace amounts of the halides remain in the ILs even after purification. Therefore, the physical and chemical properties of ILs depend on these impurities.^29,30^ Several aspects should be considered due to the presence of chloride ions in the IL. Chloride ions are considered aggressive anions in any solution where the corrosion rate of metals increases in proportion to their concentration in solution.^31−33^ Experiments on the corrosion of carbon steel in Cl^–^-containing artificial potable waters (aqueous solutions) showed that the corrosion rate at lower concentrations was small, while the corrosion potential was large. At higher concentrations, the corrosion rate increased logarithmically with decreasing corrosion potential.^34^ Thus, the presence of chloride ions in ILs influences the properties and lifetime of all devices that contain interfaces with metal electrodes and given electrolytes.

Chloride-containing IL systems can be used for applications at the industrial level. For example, dialkylimidazolium chlorides such as 1-ethyl-3-methylimidazolium chloride (EMImCl) and 1-butyl-3-methylimidazolium chloride (BMImCl) are the most popular and widely used ILs for the deposition of aluminum and its alloys. Mentioned systems have enormous potential for cathodic protection of steel.^35−37^ Another industrially important IL electrolyte class is chloroaluminates in Al ion batteries.^38,39^ Chloroaluminate ILs are the most widely employed electrolyte systems in Al ion batteries. They consist of aluminum chloride and a Lewis basic organic chloride. Chloroaluminate ILs that exhibit enhanced ion–ion interactions generally demonstrate a wider electrochemical window than ILs with weaker ion–ion interactions. These ILs function as a medium for ion transportation and as an electroactive anode material.^38^

Furthermore, chloride ions have a strong specific adsorption effect from aqueous and organic electrolytes at different electrodes.^40−43^ The adsorption activity of chloride ions depends on the solvent. This has been shown in the case of alcohols at bismuth single crystal electrodes, where the highest adsorption activity was in 2-propanol and somewhat lower in ethanol and methanol.^40,44−46^ The given trend can be explained by the decrease in the solvation effect of chloride ions and by the stronger solvent adsorption (the hydrocarbon chain in the alcohol molecules is increasing).

The specific adsorption of iodide and bromide ions from 1-ethyl-3-methylimidazolium tetrafluoroborate (EMImBF_4_) and other ILs has already been studied at the fundamental level and in applications at various surfaces.^3,49−55^ The studies have shown that the expected layered structure and the interfacial properties of the electrode|IL system are influenced by the specific adsorption of iodide and bromide ions. Bismuth single crystals are good model electrodes with proven stability and lack of surface reconstruction processes within ideal polarizability region.^47,48^ The influence of the Bi(*hkl*) crystal structure on the electrical double layer formation has been investigated.^53^ Within the potential region of specific adsorption of chloride ions, the differences between Bi(*hkl*) planes were evident, showing a pronounced dependence on the surface electronic structure as well as on the metallic characteristics of the interface. In another study, first-principles computations were performed to investigate the interfacial structure of ILs with different alkyl chains and anions that adsorbed on the Au(111) surface.^54^ In this study, the electrical double layer structure was influenced by the adsorption of chloride anion at the gold surface, which can create a local modification of the surface. At the same time, the experimental quantitative description of chloride ions’ adsorption behavior from IL is still missing.

This paper investigates the adsorption of chloride ions from EMImBF_4_ at the Bi(111) electrode. The fundamental electrochemical study will focus on the processes and the dynamics of the adsorption of chloride ions from IL. Complementing the collected data with the description of Cl^–^ adsorption at the Bi(111) single crystal electrode allows the systematic analysis of the halide ions’ adsorption phenomenon to be completed. Furthermore, these results allow us to predict how chloride impurities impact the electrochemical behavior of ILs.

## Experimental Methods

The ionic liquids used for this experimental study were 1-ethyl-3-methylimidazolium tetrafluoroborate (EMImBF_4_) (Sigma-Aldrich, for electrochemistry, purity ≥99.0%) and 1-ethyl-3-methylimidazolium chloride (EMImCl) (Sigma-Aldrich, 98%, melting point 79 °C). The studied IL mixtures (0.1, 0.5, 1, and 2 wt % of EMImCl) were prepared, and experiments were performed in a glovebox (H_2_O < 0.3 ppm, O_2_ < 0.2 ppm). EMImCl was dissolved in EMImBF_4_ by heating the mixture of ionic liquids to 70 °C and then cooled to room temperature. All experimental measurements were performed in a 3-electrode electrochemical cell, using the Bi(111) crystal with a radius of 3.8 mm as a working electrode, Pt net as a counter electrode, and AgCl-coated Ag wire as a reference electrode. The electrochemical cell for the given experiments had a volume of 4 mL. The reference electrode was connected to the working electrode compartment of an electrochemical cell through a Luggin capillary. The working electrode was polarized at a constant potential value (depending on the mixture) for a couple of hours after the electrochemical polishing to establish stable surface conditions (current density values). For cyclic voltammetry (CV) and electrochemical impedance measurements (EIS), an Autolab PGSTAT204 with an FRA32M EIS module and a Nova 1.10 software package were used. All potentials displayed in this paper were recalculated considering calibration results (in a three-electrode electrochemical cell) with a ferrocene-based system (Alfa Aesar, 99%). The formal potential of the ferrocene/ferrocenium redox couple in EMImBF_4_ was established using Pt wire as the working electrode and Ag|AgCl as the pseudo-reference electrode.

The *in situ* scanning tunneling microscopy (STM) measurements were conducted using a PicoSPM molecular imaging system in the constant current mode. The STM measurements were performed in a similar three-electrode electrochemical cell with a reduced volume (1 mL). Measuring tips were prepared through the etching of tungsten wire with KOH solution (Sigma-Aldrich, *puriss. p.a.*). The etched tungsten tips were additionally coated with insulating Apiezon Wax, which leaves only a sharp end of the tip uncovered with wax. This reduces the noise caused by the electrochemical processes occurring at the tungsten tip surface not related to the tunneling currents. The tip was introduced into the measured solution at −0.6 V. Next, after the initial stabilization of the assembled system, the surface images were collected. The postprocessing of measured STM images was done using Gwyddion data visualization and analysis software.^56^

## Results and Discussion

**Figure 1:**
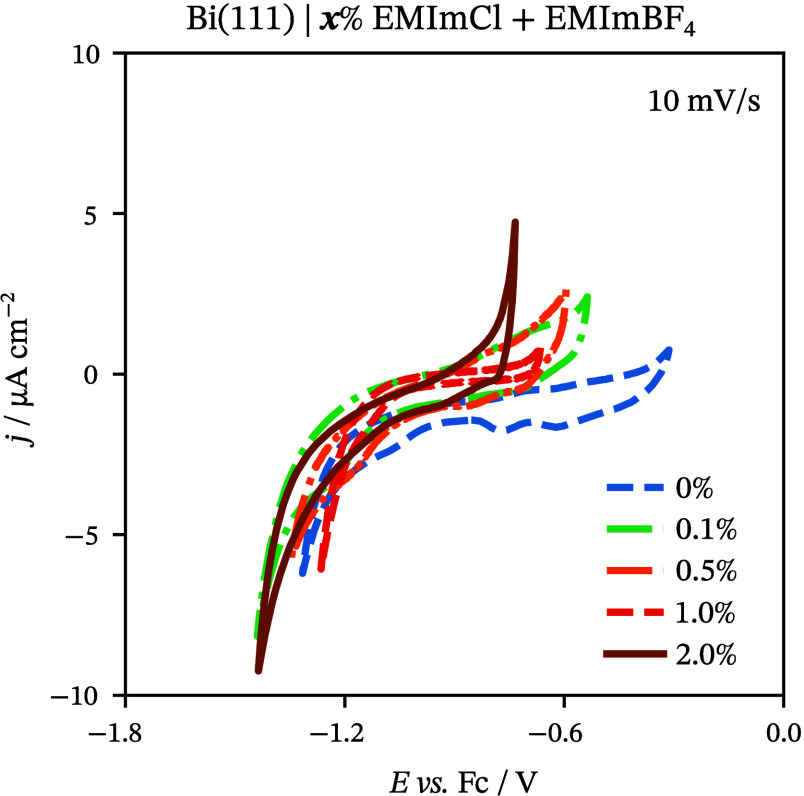
Cyclic voltammograms at a 10 mV s^–1^ scan rate for Bi(111) in EMImBF_4_ as well as in various *x* wt % EMImCl + EMImBF_4_ solutions (*x* marked in the figure).

Cyclic voltammetry was used to acquire initial qualitative information about electrochemical processes in the studied systems. CV curves express the measured current density (*j*) versus electrode potential (*E*) for Bi(111) | *x* wt % EMImCl + EMImBF_4_ in Figure 1. These dependencies were measured within the potential region of ideal polarizability (Δ*E*). The Δ*E* values are the highest for neat EMImBF_4_ and decrease with higher concentrations of EMImCl in the mixture. This results from both the specific adsorption of Cl^–^ ions at the Bi(111) surface and the earlier start of the oxidation of chloride at more positive electrode potentials. In the extended potential region, the voltammetric waves are explained by two oxidation processes of Cl^–^ at the glassy carbon, gold, and silver electrodes in contact with IL electrolyte.^57^ This work intends to study the specific adsorption of Cl^–^ ions primarily. Thus, the region of the Δ*E* value was examined.

The shape of the CV curves depends on the composition of the system, i.e., the presence and concentration of Cl^–^ ions in the electrolyte mixture. The addition of EMImCl to EMImBF_4_ increases the *j* values at more positive electrode potentials compared to the experimental data for neat EMImBF_4_ (Figure 1). This phenomenon in other organic solvents and aqueous solutions has been explained by the specific adsorption of Cl^–^ ions.^45,46^ Current results of Cl^–^ ions show similar behavior as I^–^ and Br^–^ specific adsorption processes at more positive electrode potentials at the Bi(111) electrode from EMImBF_4_.^51,52,58^ The desorption of Cl^–^ ions and structural changes at the interface affect the more negative potential region in the more concentrated mixtures.

**Figure 2:**
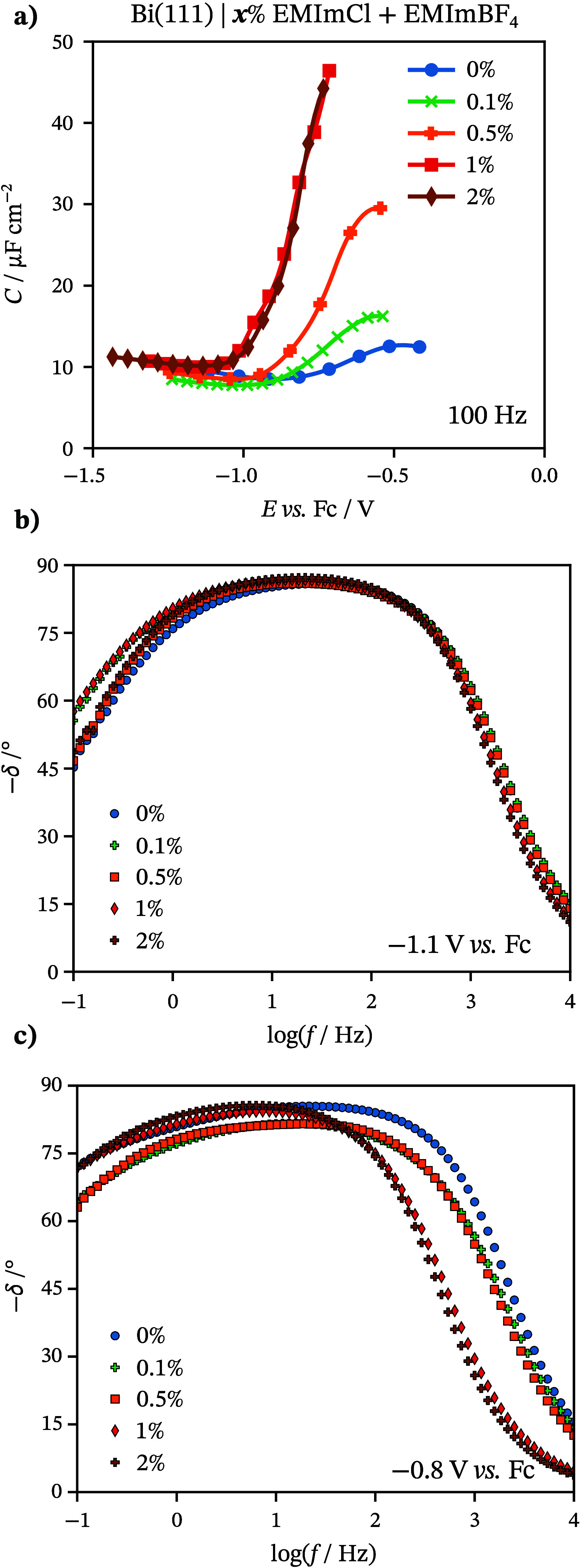
(a) Differential capacitance (*C*) vs potential *E* curves at 100 Hz. Phase angle (δ) vs log(*f*) dependencies for Bi(111) in various *x* wt % EMImCl + EMImBF_4_ solutions (*x* marked in the figure) at (b) *E* = −1.1 V and (c) −0.8 V.

The next step for explaining processes at the Bi(111) | EMImCl + EMImBF_4_ interface included electrochemical impedance spectroscopy.^59^ EIS is a powerful method for investigating the mechanisms and kinetics of electrochemical reactions. The shape of the Nyquist plot and phase angle (δ) vs periodic small-amplitude alternative current (ac) frequency (*f*) plots explain the characteristics of reactions (reversible and irreversible adsorption, mass transfer, faradaic charge transfer) occurring at the solid|electrolyte interface. The series capacitance (*C*) was calculated from Nyquist dependencies (*C* = (−*Z*″2π*f*)^−1^, where *Z*″ is the imaginary part of the impedance). *C* values depend on *E* and on the composition of the mixture (concentration of Cl^–^ ions) at constant *f* = 100 Hz, as displayed in Figure 2a. At more negative potentials, the *C* values of the studied mixtures are not different from those of neat EMImBF_4_. This indicates that the electrical double layer innermost structure is not strongly influenced by the presence of Cl^–^ ions in the mixture at these potentials. Thus, the capacitance and so-called IL contact layer are mostly influenced by imidazolium cations. The δ vs ac *f* dependencies at this *E* region also support the previous findings (Figure 2b). The estimated δ values are almost independent of the composition of the IL mixtures in the whole ac *f* range at *E* = −1.1 V. Almost ideal capacitive behavior (δ values are lower than −80°) can be seen within the wide range of ac *f* values (1 Hz < *f* < 500 Hz) for all measured concentrations. However, in lower ac *f* (*f* < 1 Hz), the mixed kinetic behavior (adsorption and diffusion limiting character of the processes) prevails, but mixtures with higher Cl^–^ concentration have somewhat lower δ values, indicating that adsorption in mixed kinetics limited processes has a higher proportion. Thus, the nature of the limiting steps of the interfacial processes is similar at various EMImCl concentrations in the case of Bi(111) | *x* wt % EMImCl + EMImBF_4_.

The specific adsorption of Cl^–^ ions at the Bi(111) surface occurs at more positive electrode potentials, evident from higher *C* values compared to neat EMImBF_4_ (Figure 2a). There is a limiting Gibbs adsorption effect of Cl^–^ ions for more concentrated mixtures (2 wt %), as the *C* values do not increase further. This phenomenon is well described in aqueous solutions and organic solvents.^40,45,46^ In Figure 2c, the shift of δ at a higher ac *f* region (*f* > 100 Hz) shows that the electrical double layer charging is slower in more concentrated mixtures. This can be explained by a slow specific adsorption process of Cl^–^ and the formation of a dense ordered chloride layer at the electrode surface. The mixed kinetic behavior (adsorption and diffusion limiting character of the processes) describes the processes at *f* < 100 Hz.

**Figure 3:**
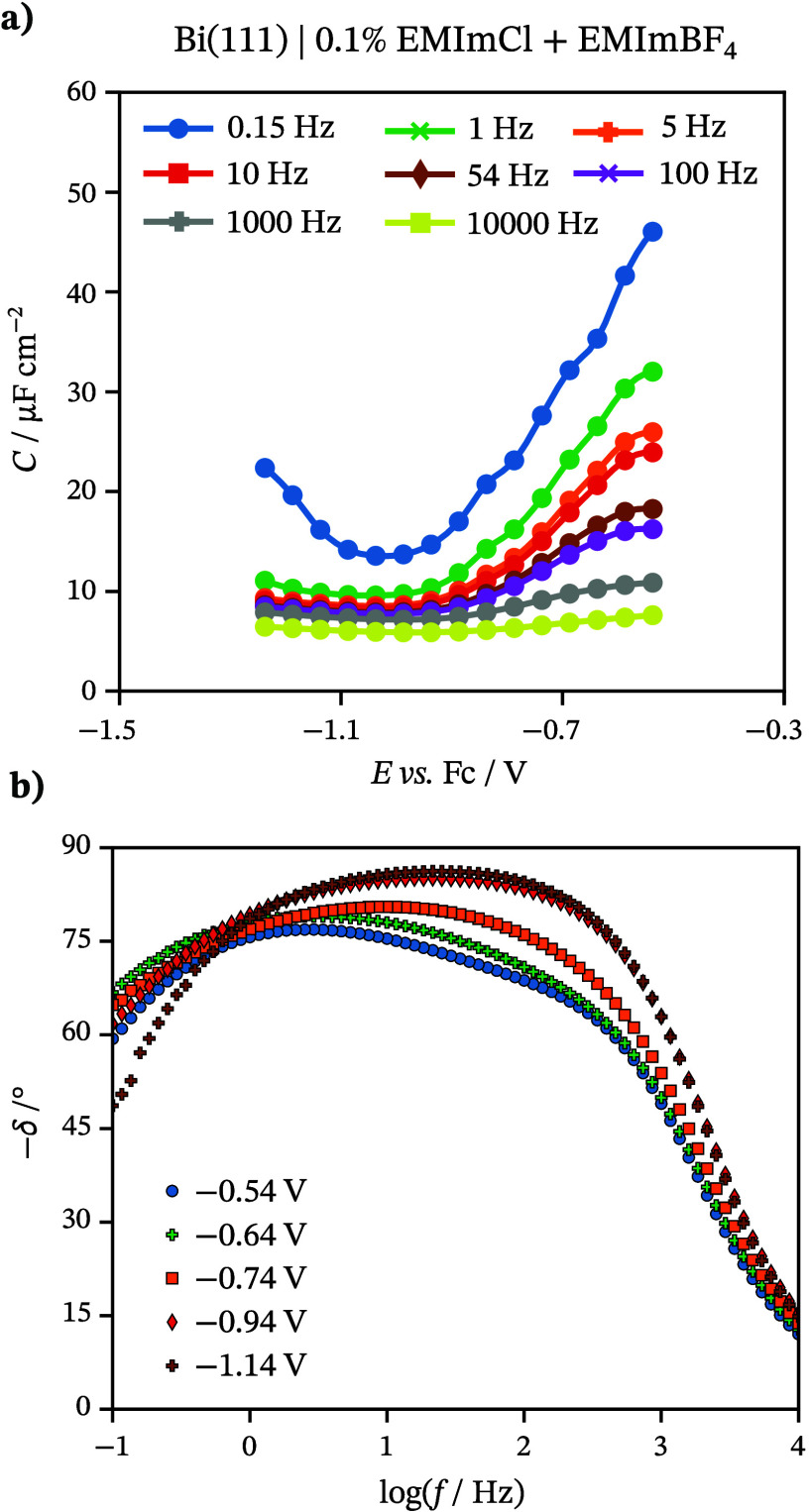
(a) Differential capacitance (*C*) vs potential (*E*) curves at various frequencies (marked in the figure). (b) Phase angle (δ) vs log(*f*) dependencies for Bi(111) in 0.1 wt % EMImCl + EMImBF_4_ solution.

The *C* does not depend significantly on *E* at *f* > 100 Hz, while the alteration of *f* at moderate and low ac *f* region leads to the increase of *C* values at the more positive *E* region (Figure 3a). The high-viscosity values of EMImBF_4_ and the mixtures can explain this. Because of high viscosity, all processes, including specific adsorption of Cl^–^ ions, are slow and prevail in *C* values only within moderate and low ac *f* regions. This is confirmed in previous studies using IL-based systems.^52,60^ The increase of *C* values at the limits of the ideal polarizability region in the case of the low *f* (*f* < 1 Hz) is caused by the specific adsorption of anions at less negative potentials and pseudocapacitive behavior (desorption of chloride ions) of the system at more negative potentials. In Figure 3b, within a high ac *f* region (*f* > 500 Hz), the electrical double layer charging is faster (shifted to higher ac *f* values) within the *E* region where the adsorption of Cl^–^ does not appear (*E* < −0.9 V). In this *E* region, the δ values are lower than −82° within a wide *f* region (1 Hz < *f* < 500 Hz), describing nearly ideal capacitive behavior. However, within the *E* region of Cl^–^ adsorption (*E* > −0.9 V), the mixed kinetic behavior (adsorption and diffusion limiting character of the processes) is limiting within the same ac *f* region. In the low ac *f* range (*f* < 1 Hz), lower δ values indicate that the processes are limited by adsorption, diffusion, and charge transfer processes.

**Figure 4:**
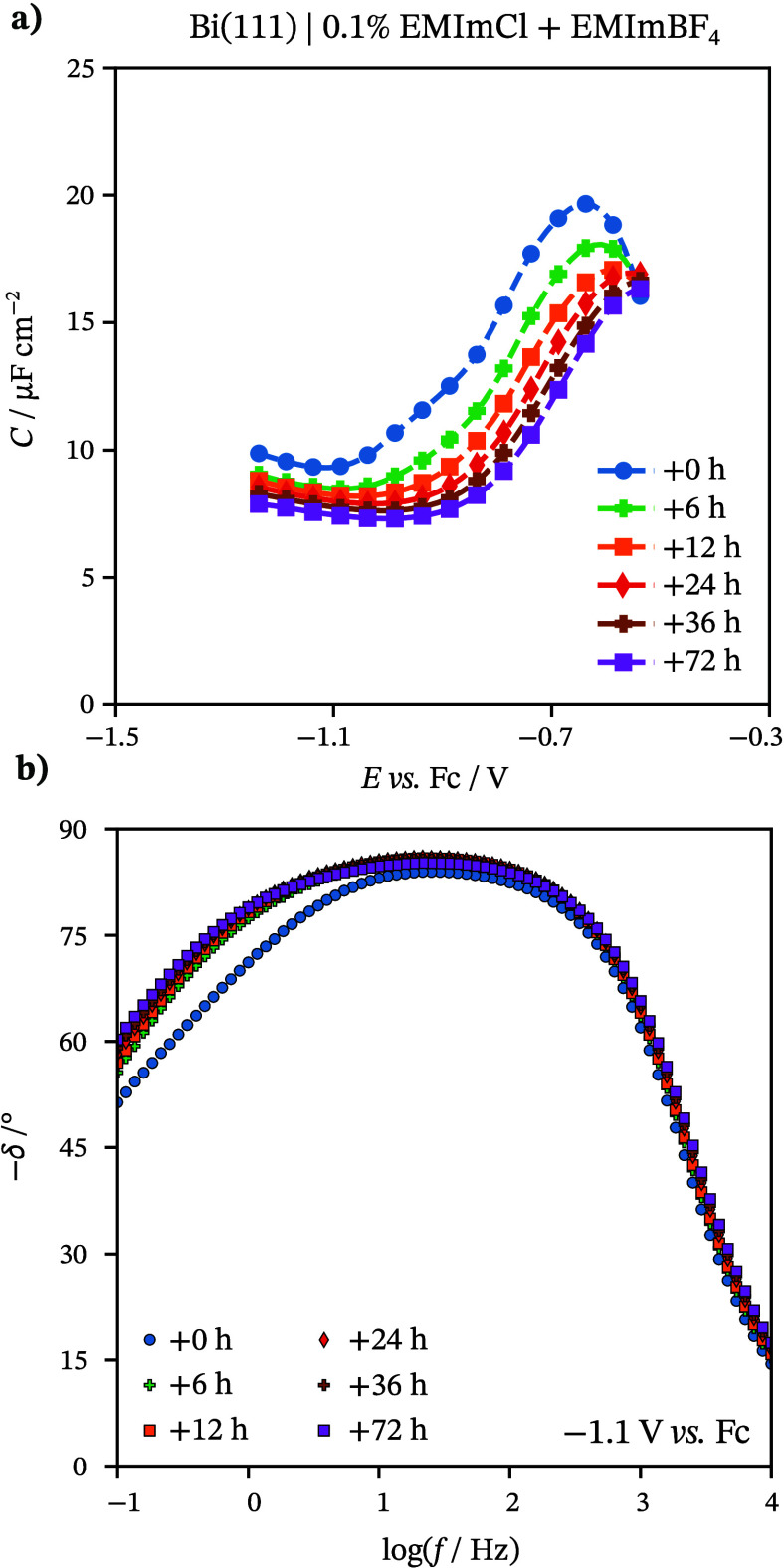
(a) Differential capacitance (*C*) vs potential (*E*) curves over various times at 100 Hz (marked in the figure). (b) Phase angle (δ) vs log(*f*) dependencies for Bi(111) in 0.1 wt % EMImCl + EMImBF_4_ solution.

The equilibration of the interface and the formation of a stable adsorbed layer of Cl^–^ ions are lengthy process timewise. *C* values decrease in time, reaching a stable state after 36 h (Figure 4a). Kinetic analysis of these systems supports the findings as δ values decrease in time in the low ac *f* range (Figure 4b). The total rate of mixed kinetic processes is limited by the diffusion-like and adsorption steps. In higher ac *f* values, there is no remarkable time dependence, which indicates a very slow adsorption process of Cl^–^ ions.

**Figure 5:**
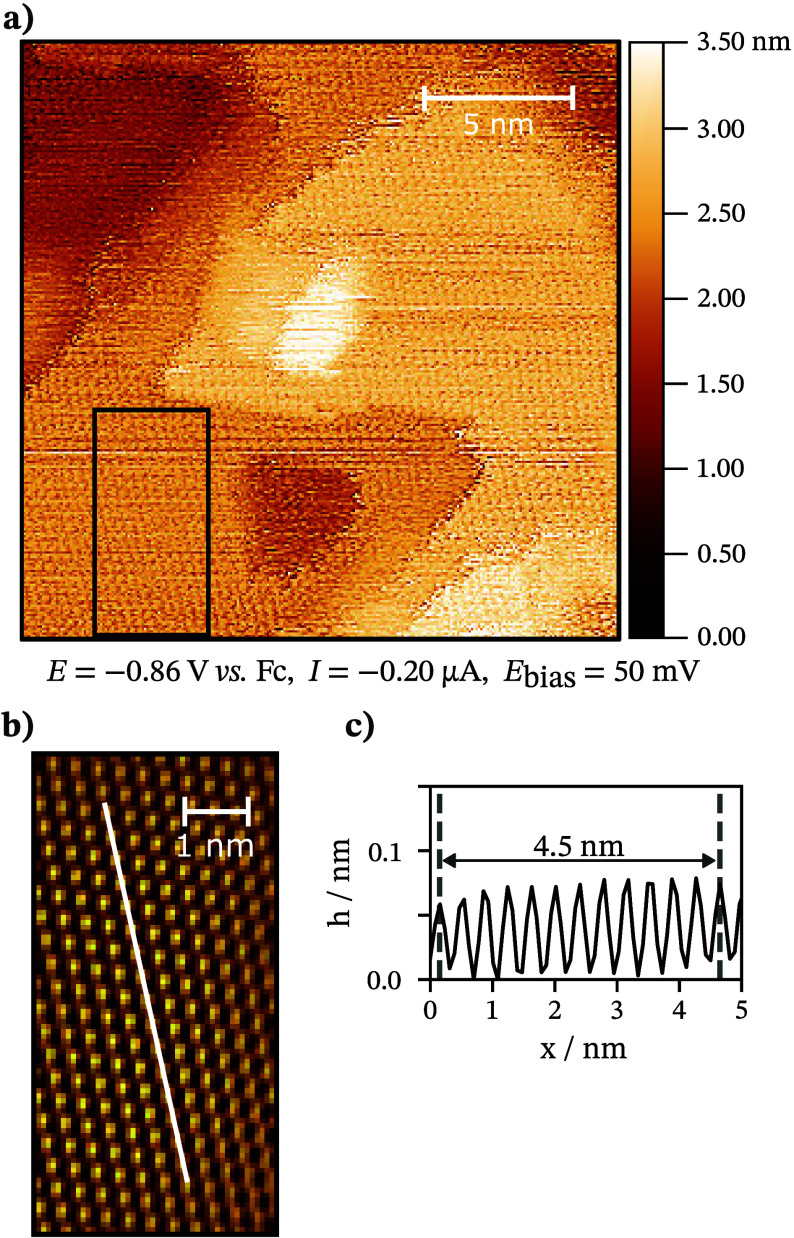
(a) *In situ* STM image of Bi(111) | 0.5 wt % EMImCl + EMImBF_4_ interface at potential *E* = −0.86 V. (b) A Fourier transform filtered image of the surface. (c) Height profile of marked line on filtered STM image for the determination of the distance between the atoms. The image was measured after 8 h since the assembly of the electrochemical cell.

The *in situ* STM measurements provide insights into the interfacial processes in real space. A highly ordered structure was visualized at *E* = −0.86 V (see Figure 5a), which is in agreement with the beginning of the Cl^–^ adsorption process, displayed in Figure 2a. The regular structure was denoised using two-dimensional Fourier transform filtering. The denoised image, shown in Figure 5b, consists of round-shaped elements separated by 3.75 Å. The distance between the elements is in good agreement with twice the effective ionic radii of Cl^–^ ion (1.81 Å^61^), while the two Bi atoms of Bi(111) surface in the same layer are separated by 4.54 Å.^62^ Thus, we consider the highly ordered structure to consist of adsorbed Cl^–^ ions. The adsorbed Cl^–^ ions form a dense adlayer, similar to I^–^^63^ and trifluoromethanesulfonate (OTf^–^)^64^ ions at Bi single-crystal surfaces in IL electrolytes.

**Figure 6:**
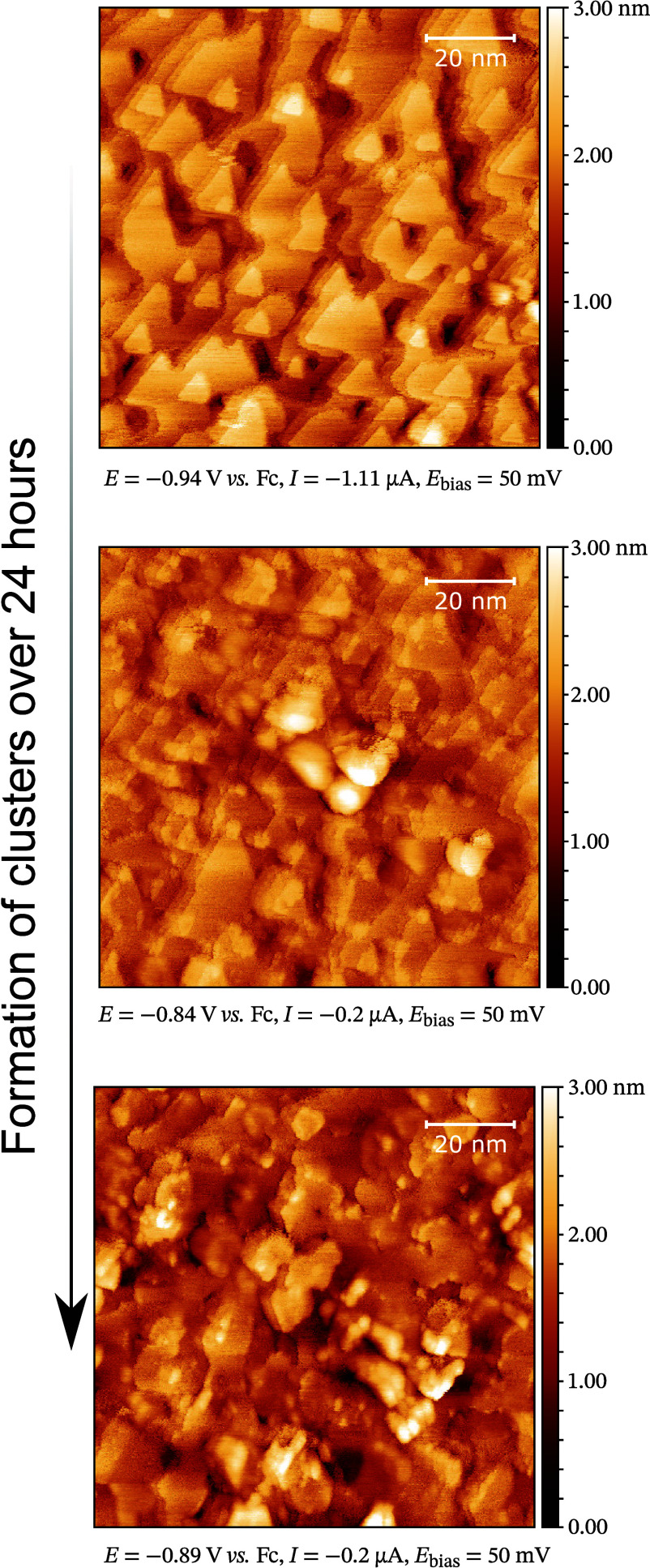
*In situ* STM images of Bi(111) | 0.5 wt % EMImCl + EMImBF_4_ illustrating the formation of clusters on the Bi(111) surface.

The changes in the interfacial properties over time, described by the EIS measurements, are also visible with *in situ* STM. After the electrochemical polishing, the imaged surface consists of terraces with triangular shapes, characteristic of electrochemically polished Bi(111) surface.^65^ During the 24 h polarization period, the roughening of the surface due to the formation of superstructures took place (Figure 6). The formation of clusters first only occurred on several defect areas or step edges, while after some time, the growth of existing clusters and the formation of new ones were imaged. Previously, a similar roughening process has been imaged in the case of the Bi(111) | EMImI + EMImBF_4_ system, where it was related to the selective anodic dissolution of the flat Bi(111) surface.^63^ Although possible, we relate the formation of clusters on the Bi(111) surface and the changes of capacitance as well as phase angle over time to the very slow formation of equilibrium interfacial structures, involving the specific adsorption of Cl^–^ as well as the coadsorption of EMIm^+^ cations. Furthermore, the anodic dissolution of the surface and roughening of the electrode structure have been shown to increase the capacitance of Cd(0001)|EMImI + EMImBF_4_, effectively increasing the electrode’s surface area.^66^

## Conclusions

The interfacial structure and properties of the electrode|ionic liquid systems are crucial for developing fundamental understanding and various applications. To study the electrochemical effect in the limits of adsorption of chloride ions at the Bi(111) electrode, the mixtures of 1-ethyl-3-methylimidazolium tetrafluoroborate and 1-ethyl-3-methylimidazolium chloride were electrochemically characterized by using cyclic voltammetry and electrochemical impedance spectroscopy. The characterization of interfacial processes in real space was visualized by *in situ* STM. Within the potential region of specific adsorption of Cl^–^, capacitance values increased significantly compared to neat EMImBF_4_ and were limited by Gibbs adsorption effect at high Cl^–^ concentration. The equilibration of the interface and the formation of a stable adsorbed layer of Cl^–^ ions is a lengthy process timewise. *C* values decrease in time, reaching a stable value after 36 h. Phase angle values confirmed that the kinetics of these processes is limited by the adsorption kinetic step in a wide range of alternative current frequencies. The findings are supported by *in situ* STM images, where a highly ordered structure was visualized. The structure consists of round-shaped elements separated by 3.75 Å. The distance between the elements is in good agreement with twice the effective ionic radii of the Cl^–^ ion (1.81 Å). Over time, the formation of clusters and superstructures on the Bi(111) surface was imaged.
